# Pregnancy and post-partum tuberculosis; a nationwide register-based case–control study, Denmark, 1990 to 2018

**DOI:** 10.2807/1560-7917.ES.2022.27.12.2100949

**Published:** 2022-03-24

**Authors:** Anne Christine Nordholm, Camilla Hiul Suppli, Anders Norman, Claus Thorn Ekstrøm, Pia Ertberg, Anders Koch, Troels Lillebaek, Aase Bengaard Andersen

**Affiliations:** 1 International Reference Laboratory of Mycobacteriology, Statens Serum Institut, Copenhagen, Denmark; 2 Section of Biostatistics, Department of Public Health, University of Copenhagen, Copenhagen, Denmark; 3 Department of Obstetrics and Gynaecology, Herlev/Gentofte Hospital, Herlev, Denmark; 4 Department of Infectious Disease Epidemiology and Prevention, Statens Serum Institut, Copenhagen, Denmark; 5 Global Health Section, Department of Public Health, University of Copenhagen, Copenhagen, Denmark; 6 Department of Infectious Diseases, Rigshospitalet Copenhagen University Hospital, Copenhagen, Denmark

**Keywords:** TB control, risk factor, screening, public health management, antenatal care

## Abstract

**Background:**

Pregnancy increases the risk of tuberculosis (TB), however, data on TB epidemiology in pregnant women are limited.

**Aim:**

To guide possible interventions, we analysed risk factors for TB in pregnant and post-partum women.

**Methods:**

We conducted a nationwide retrospective register-based case–control study from January 1990 to December 2018 in Denmark. Cases were women diagnosed with TB during their pregnancy or in the post-partum period. We selected two control groups: pregnant or post-partum women without TB, and non-pregnant women with TB. Differences were assessed by chi-squared or Fisher’s exact test. Risk factors for TB were identified through logistic regression and estimated by odds ratio (OR).

**Results:**

We identified 392 cases, including 286 pregnant and 106 post-partum women. Most were migrants (n = 366; 93%) with a shorter median time spent in Denmark (2.74 years; interquartile range (IQR): 1.52–4.64) than non-pregnant TB controls (3.98 years; IQR: 1.43–8.51). Cases less likely had a Charlson comorbidity index ≥ 2compared with non-pregnant TB controls (p < 0.0001), and had no increased risk of severe disease (p = 0.847). Migrants from other World Health Organization regions than Europe, especially Africa (OR: 187; 95%CI: 125–281) had persistently higher odds of TB.

**Conclusions:**

In Denmark, the risk of TB in pregnant and post-partum women is increased in migrant women who have stayed in the country a median time of approximately 3 years. We recommend increased focus on TB risk during pregnancy and suggest evaluating targeted TB screening of selected at-risk pregnant women to promote early case finding and prevent TB among mothers and their newborn children.

## Introduction

The true global burden of tuberculosis (TB) during pregnancy is not known, but it is estimated that there are more than 200,000 cases per year [1]. Early diagnosis of TB among pregnant women is essential because TB poses a risk of serious illness not only for the pregnant woman but also in particular for the newborn child [2-5]. Pregnancy increases the risk of progressing from TB infection (TBI) to active TB [6] by different hormonal and immunological changes [7-9]. This shift leads to impaired cellular immunity and thus increased susceptibility to certain infections such as TB [7,10]. A recent study from Sweden found an increased risk of TB during pregnancy and the post-partum period among migrants from TB high-incidence countries [11], in line with findings from the United Kingdom (UK) [12]. Interestingly, screening of eligible pregnant migrants from countries with high TB incidence has been implemented in the Swedish antenatal care programme with a high yield of both TBI and TB case detection [13].

TB among pregnant and post-partum women has not been highly prioritised in TB research and TB control programmes [5,14]. Across Europe, there are very variable policies for screening migrants and other TB risk groups, and only few countries have specific guidelines addressing pregnancy and TB risk [15,16]. In Denmark, there are limited data on pregnancy and TB, and there are no guidelines on the control and management of TB in pregnant migrants from high-incidence countries. In this nationwide study, we describe women who are diagnosed with TB during pregnancy and the post-partum period in Denmark and analyse risk factors, in order to guide future public health policies.

## Methods

### Setting

In Denmark, healthcare is free for all residents, including antenatal care, TB diagnostics and treatment. It is mandatory to notify TB to the health authorities and data are systematically registered in the national TB Surveillance Register (TBSR), centrally hosted at the Department of Infectious Diseases Epidemiology and Prevention, Statens Serum Institut (SSI). All TB diagnostics are centralised at the International Reference Laboratory of Mycobacteriology (IRLM), SSI. Residents in Denmark are assigned a unique central personal identification (CPR) number in the Civil Registration System (CRS). This CPR number can be used to individually track and link information in national registries and databases.

### Design and study population

This was a nationwide retrospective register-based case–control study conducted in Denmark from January 1990 through December 2018. Cases were women diagnosed with TB during pregnancy or during the post-partum period, who were compared with two control groups; pregnant or post-partum women from the general population without TB, and non-pregnant/non-post-partum women with TB.

### Data generation

TB cases were identified in the TBSR, from which information on ethnicity, country of origin, country of infection, and sources of infection was extracted. To avoid duplicate TB cases, a case could only count once during a 12-month period in accordance with World Health Organization (WHO)/European Centre for Disease Prevention and Control (ECDC) guidelines [17]. The pregnant TB cases were defined as women who had been diagnosed with TB up to 9 months before giving birth. Post-partum TB cases were defined as women who were diagnosed with TB up to 12 months following delivery. The National Patient Register (NPR) was used to identify pregnant and post-partum cases as this registry contains diagnoses codes and time of diagnoses according to the International Classification of Diseases (ICD) 8 (1989–1993) and ICD 10 (after 1993). From the NPR, we extracted information on hospitalisations/outpatient hospital visits and length of admission in days. In addition, diagnoses codes were used to calculate the Charlson comorbidity index (CCI) [18]. We obtained information on culture-verified TB cases from the IRLM, SSI. From the CRS, we obtained information on date of birth and death, sex, place of residency, and date of immigration into Denmark for migrants. From the National Registry of Causes of Deaths, we extracted information on death causes.

**Figure 1:**
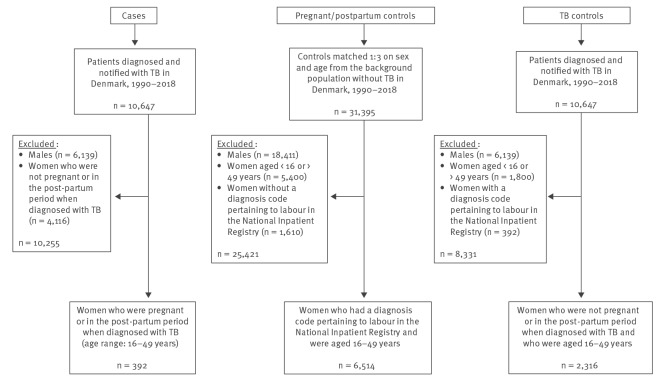
Study population for a nationwide register-based case–control study to assess risk factors for tuberculosis during pregnancy and the post-partum period, Denmark, 1990–2018 (n = 53,229)

The generation of the two control groups is illustrated in Figure 1. The first control group ‘pregnant/post-partum controls’ was generated from a preselected control group containing controls without TB from the general population, matched on sex and age to all TB cases. From this preselected control group, we included all women who had a diagnosis code pertaining to labour (ICD8: 650–662, ICD10: DO80–DO84) and who were in the same age range as cases (16–49 years).

TB: tuberculosis.

The second control group ‘TB controls’ was generated from the TB population and defined as women aged 16–49 years who had not given birth up to 9 months after the date of a TB diagnosis and who had not been diagnosed with TB up to 12 months after delivery.

From the Statistics Denmark Database, containing most nationwide registries, we extracted information of family structure, highest educational attainment, employment status and income for cases and controls. Data from registers and databases were linked by the CPR number and subsequently anonymised. Data were handled by secured individual access to Statistics Denmark.

### Definitions

In this study, cases and controls were categorised as either migrants or Danes, according to their geographical area of origin. Danes were defined as being born in Denmark with both parents also born in Denmark. Migrants were defined as persons born outside of Denmark or persons with one or both parents born outside of Denmark. Denmark and Greenland are constituents of the Kingdom of Denmark, however the incidence of TB differs in these two parts, as the incidence is much higher in Greenland (88 per 100,000 population per year) [19], than in Denmark (5 per 100,000 population per year) [20]. As such, persons born in Greenland, or with one or both parents born in Greenland were classified as migrants for the purpose of the study.

Countries of origin were stratified into the six WHO regions: European Region, Region of the Americas, African Region, Eastern Mediterranean Region, South-East Asian Region and Western Pacific Region. Generally, and in accordance with WHO guidelines [21], pulmonary TB (PTB) was defined as TB involving the lung parenchyma and/or tracheobronchial tree including miliary TB and mixed PTB/extrapulmonary TB (EPTB). EPTB was defined as TB involving organs other than the lungs. Severe TB was defined as having at least two of the following conditions: multiple-organ disease, culture-positive disease, or a long hospital admission of > 10 days. To serve as proxies for socioeconomic status (SES) we used highest attained education, employment status and yearly income.

### Statistical analyses

Categorical data were described by numbers and percentages. For data comparisons, differences were assessed by chi-squared test or Fisher’s exact test where appropriate. Continuous variables were presented as medians and 25–75% interquartile range (IQR) and compared by the non-parametric Wilcoxon rank sum test. 

Risk factors for TB were assessed among cases and pregnant/post-partum controls. We estimated the risk of TB by logistic regression analyses calculating odds ratio (OR) with 95% confidence interval (CI) using Wald test. Initially we included the following variables: outcome was TB, and exposures were categories according to origin (Danes/migrants; WHO region of origin), years since immigration, CCI, number of persons in household, education, employment and income. However, due to a high number of cases from Africa with missing data for education, the final model was fitted to only include WHO region of origin, CCI, number of persons in household, employment status (employed/unemployed), and income level (low (< EUR 15,000 per year)/high (≥ EUR 15,000 per year)). We tested for interactions and collinearity, which did not affect the estimates. 

Risk factors for severe TB were estimated by comparing cases with TB controls; the outcome was severe TB, exposure variables were category (Danes/migrants), CCI, employment status and income level. Data management and statistical analyses were carried out in SAS version 9.4.

### Ethical statement

The study was approved by the Danish Data Protection Agency (J.no 19/04240). No further ethical approval is required in Denmark for registry-based research studies. Data were analysed anonymously and assessed online in the Statistics Denmark’s research database.

## Results

### Basic demography, socioeconomic status and comorbidities

**Figure 2:**
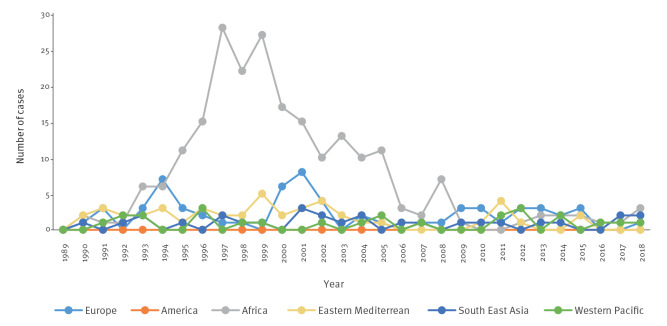
Distribution of annual numbers of women diagnosed with tuberculosis while pregnant or in the post-partum period (cases), stratified by their World Health Organization region of origin, Denmark, 1990–2018 (n = 392)

We identified 392 women who were pregnant (n = 286) or in the post-partum period (n = 106) when diagnosed with TB in Denmark from 1990 to 2018. The majority were migrants (n = 366; 93%), predominantly from Africa (n = 228; 58%) (Table 1). Among migrants, women from Somalia accounted for 54% (n = 199), followed by Pakistan (n = 34; 9%), the Philippines (n = 13; 4%), Vietnam (n = 10; 3%), and Thailand (n = 9; 2%). During the study period, the distribution of the women’s countries of origin varied (Figure 2). From 1993 to 2006, the largest proportion of migrant cases was constituted by African women (n = 202), especially from Somalia (186/202; 92%). The distribution according to geographical area of origin was similar among TB controls, but different from the pregnant/post-partum-controls, of whom 89% were Danes and of whom the majority of migrants was from other European countries (Table 1). Among migrants, the median time in Denmark since immigration was substantially shorter among TB cases (2.74 years; IQR: 1.52–4.64) compared with TB controls (3.98 years; IQR: 1.43–8.51). Generally, cases and TB controls were of significantly lower education, were less employed and had a lower yearly income than pregnant/post-partum controls (Table 1). In contrast to the cases and pregnant/post-partum controls, TB controls more frequently lived in crowded conditions (> 5 persons in the household). The majority of cases had no noteworthy comorbidities comparable to pregnant/post-partum controls but were considerably different from the TB controls who had higher CCI (Table 1). Diabetes, HIV and hepatitis were more common among cases and TB controls than among pregnant/post-partum controls (Table 1).

**Table 1:** Characteristics of women diagnosed with tuberculosis while pregnant or in the post-partum period (cases) compared with pregnant/post-partum controls and tuberculosis controls, Denmark, 1990–2018 (n = 9,222)

Characteristic	Cases		Pregnant/post-partum controls		TB controls		p^a^
	n = 392	%	n = 6,514	%	n = 2,316	%	
**Age group (years)**							
15–24	107	27	1,287	20	481	21	< 0.0001
25–34	230	59	2,697	41	846	37	
35–50	55	14	2,530	39	981	42	
**Total^b^**	**392**	**NA^c^**	**6,514**	**NA^c^**	**2,308**	**NA^c^**	**NA**
**Category^d^**							
Danes^d^	26	7	5,799	89	571	25	< 0.0001
Migrant^d^	366	93	715	11	1,745	75	
**Total^b^**	**392**	**NA^c^**	**6,514**	**NA^c^**	**2,316**	**NA^c^**	**NA**
**Country or WHO region of origin**							
Denmark	26	7	5,799	89	571	25	< 0.0001
European Region	34	9	357	5	406	18	
Region of the Americas	1	0	13	0	15	1	
African Region	228	58	59	1	547	24	
Eastern Mediterranean Region	49	13	190	3	315	14	
South-east Asian Region	26	7	37	1	220	10	
Western Pacific Region	28	7	58	1	241	10	
**Total^b^**	**392**	**NA^c^**	**6,513**	**NA^c^**	**2,315**	**NA^c^**	**NA**
**Time from immigration to Denmark until TB diagnosis (years)^e^**							
0–2	200	55	0	0	831	47	< 0.0001
3–5	106	29	0	0	308	18	
6–10	39	11	0	0	308	18	
≥ 11	21	6	0	0	298	17	
**Total^b^**	**366**	**NA^c^**	**0**	NA^c^	**1,745**	**NA^c^**	**NA**
**Number of persons in household**							< 0.0001
1	4	1	534	8	306	13	
2–3	111	28	3,158	48	962	42	
4–5	153	39	2,484	38	606	26	
> 5	122	31	338	5	432	19	
**Total^b^**	**390**	**NA^c^**	**6,514**	**NA^c^**	**2,306**	**NA^c^**	**NA**
**Educational level**							
Elementary school	180	53	2,477	39	1,027	55	< 0.0001
High school	65	19	1,283	20	246	13	
Short higher	68	20	1,623	25	412	22	
University degree	27	8	997	16	180	10	
**Total^b^**	**340**	**NA^c^**	**6,380**	**NA^c^**	**1,865**	**NA^c^**	**NA**
**Employment**							
Employed	44	11	4,370	67	576	25	< 0.0001
Unemployed	9	2	329	5	108	5	
On leave	11	3	228	4	74	3	
Education	5	1	841	13	217	9	
Disability pension	3	1	135	2	202	9	
Cash benefit recipient	230	59	402	6	693	30	
Unknown/other	90	23	209	3	446	19	
**Total^b^**	**392**	**NA^c^**	**6,514**	**NA^c^**	**2,316**	**NA^c^**	**NA**
**Yearly income (EUR)**							
< 15,000	364	93	2,657	41	1,876	81	< 0.0001
15,000–29,000	15	4	1,745	27	237	10	
30,000–34,000	10	3	1,443	22	141	6	
≥ 45,000	3	1	669	10	62	3	
**Total^b^**	**392**	**NA^c^**	**6,514**	**NA^c^**	**2,316**	**NA^c^**	**NA**
**Charlson comorbidity index**							
0	292	75	5,006	77	1,363	59	< 0.0001
1	57	15	828	13	395	17	
≥ 2	43	10	680	10	558	24	
**Total^b^**	**392**	**NA^c^**	**6,514**	**NA^c^**	**2,316**	**NA^c^**	**NA**
**Comorbidities**							
Diabetes	18	5	183	3	144	6	< 0.0001
HIV	8	2	4	0	94	4	
HBV	10	3	16	0	64	3	
HCV	1	0	21		63	3	
**Total^b^**	**392**	**NA^c^**	**6,514**	**NA^c^**	**2,316**	**NA^c^**	**NA**

HBV: hepatitis B virus; HCV: hepatitis C virus; HIV: human immunodeficiency virus; NA: not applicable; TB: tuberculosis; WHO: World Health Organization.

^a^ Differences between cases and controls were estimated by Chi-squared test or Ficher’s exact test for categorical variables.

^b^ Except for the subsection entitled ‘Comorbidities’, the total of a column in each horizontal subsection represents the sum of the numbers in that column. This sum is used as the denominator for the percentages presented in the next column. Because totals are based only on the numbers of persons with available data, they may differ from the totals provided in the Table header, for the overall columns. For the subsection entitled ‘Comorbidities’ the total of a given column does not represent the sum of numbers in that column, but only indicates the total used for the percentages in the next column.

^c^ The totals of percentages, which are not presented, can be different from 100, because of rounding issues.

^d^ The category is based on the geographical area of origin. Because Greenland has a different TB epidemiology than Denmark, Greenlanders (comprising people born in Greenland or with at least one parent born in Greenland) are classified as migrants in the study. Among cases, seven women were Greenlanders. There were no Greenlanders among pregnant/post-partum controls, whereas there were 210 Greenlanders among the TB controls.

^e^ Only calculated for immigrants having a migration date in the Civil Registration System.

### Disease site and severity

More TB controls had PTB and were smear positive compared with cases, however there were no difference in the proportion of severe TB cases in the two groups (Table 2). There were no significant differences in the proportion of cases and controls being hospitalised (50% vs 52% respectively; p = 0.097) or the median lengths of admission (13 days (IQR: 7–20) vs 12 days (IQR: 7–19) respectively; p = 0.88). Mortality was highest among TB controls, though few died from TB (Table 2 ).

**Table 2:** Tuberculosis characteristics in women diagnosed while pregnant or in the post-partum period (cases) compared with tuberculosis controls, Denmark, 1990–2018 (n = 2,708)

TB characteristic	Cases n = 392		TB controls n = 2,316		p^b^
	Number	%^a^	Number	%^a^	
Pulmonary TB	205	52	1,555	67	< 0.0001
Smear-positive pulmonary TB	69	34	706	45	0.0015
Culture positive	313	80	1,769	76	0.1323
INH mono resistance	21	5	100	4	0.3570
MDR	2	0	10	0	0.8288
Infected abroad	316	81	1,445	62	< 0.0001
Severe TB^c^	111	28	618	27	0.847
Number of deaths	3	1	236	10	< 0.0001
TB related deaths	0	0	34	1	< 0.0001

INH: isoniazide; MDR: multidrug resistance; TB: tuberculosis.

^a^ Except for the smear-positive pulmonary TB, the denominators for the percentages in the cases and TB controls columns are the total number of cases (n = 392) or TB controls (n = 2,316) respectively. For the smear-positive pulmonary TB, the denominators are the number of pulmonary TB cases (n = 205) or controls (n = 1,555).

^b^ Differences between cases and controls were tested by Chi-square/Fisher’s exact test.

^c^ Severe TB was defined as having at least two of the following conditions: multiple-organ disease, culture-positive disease or hospital admission of > 10 days.

### Risk factors

Univariable logistic regression showed high odds of TB among migrants from outside the European Region, living in crowded environments (> 5 per household), with low education, who were unemployed or who had low income (Table 3 ). Due to a high number of missing data for the variable education (n = 186), especially among cases from Africa, this variable was removed when fitting the final model. In the fitted multivariable logistic regression model, we adjusted for persons in household, CCI, employment and income, and found extremely high odds for TB among migrants from Africa (OR: 187) compared with European origin (including Danes and Greenlanders) (Table 3). Being unemployed and having a low yearly income were significantly associated with TB. The multivariable regression model assessing risk of severe TB showed no significant associations for category (Danes/migrants), CCI, employment status or income (Table 4).

**Table 3:** Associations between tuberculosis and origin, socioeconomic status, and comorbidity among women diagnosed with tuberculosis while pregnant or in the post-partum period (cases) and pregnant/post-partum controls, Denmark, 1990–2018 (n = 6,906)

Characteristic	Univariable logistic regression			Characteristic	Multivariable logistic regression^a^		
	OR	95% CI	p^b^		OR	95% CI	p^b^
**WHO region of origin (n = 6,904**)			**< 0.0001**	**WHO region of origin (n = 6,904)**			**< 0.0001**
European Region	Ref	NA	Ref	European Region	Ref	NA	Ref
Region of the Americas	7.82	1.02–61.3	0.05	Region of the Americas	7.14	0.88–58.1	0.0761
African Region	396	270–581	< 0.0001	African Region	187	125–281	< 0.0001
Eastern Mediterranean Region	26.5	17.7–39.6	< 0.0001	Eastern Mediterranean Region	11.8	7.56–18.4	< 0.0001
South-East Asian Region	72.1	41.1–126	< 0.0001	South-East Asian Region	50.2	27.7–91.0	< 0.0001
Western Pacific Region	49.5	29.5–83.1	< 0.0001	Western Pacific Region	36.9	20.9–62.3	< 0.0001
**Number of persons in household (n = 6,904)**			**< 0.0001**	**Number of persons in household^c^ (n = 6,904)**			**0.0009**
1	Ref	NA	Ref	1–4	Ref	NA	Ref
2–3	4.69	1.72–12.8	0.0025				
4–5	8.22	3.03–22.3	< 0.0001				
> 5	48.2	17.6–131	< 0.0001	≥ 5	1.94	1.31–2.84	0.0009
**Educational level (n = 6,720**)			**< 0.0001**	**Educational level (n = 6,720)**			**ND**
Elementary school	Ref	NA	Ref	Elementary school	ND	ND	ND
High school	0.70	0.52–0.93	0.0153	High school	ND	ND	ND
Short higher	0.58	0.43–0.77	< 0.0001	Short higher	ND	ND	ND
University degree	0.37	0.25–0.56	< 0.0001	University degree	ND	ND	ND
**Employment status**			**< 0.0001**	**Employment status**			**0.0151**
Employed	Ref	NA	Ref	Employed	Ref	NA	Ref
Not-employed	16.1	11.7–22.2	< 0.0001	Not employed	2.04	1.15–3.63	0.0151
**Yearly income (EUR)**			**< 0.0001**	**Yearly income (EUR)**			**0.0005**
< 15,000	Ref	NA	Ref	< 15,000	Ref	NA	Ref
≥ 15,000	0.05	0.03–0.07	< 0.0001	≥ 15,000	0.31	0.16–0.59	0.0005
**Charlson comorbidity index**			**0.2929**	**Charlson comorbidity index**			**0.9177**
0	Ref	NA	Ref	0	Ref	NA	Ref
≥ 1	0.86	0.64–1.14	0.2929	≥ 1	0.98	0.64–1.50	0.9177

CI: confidence interval; NA: not applicable; ND: not determined; OR: odds ratio; Ref: reference; WHO: World Health Organization.

In case of missing data for a variable, the number of cases with known information is indicated in parentheses.

^a^ All the listed variables, except education were adjusted for in the final multivariable model.

^b^ Wald test.

^c^ The variable ‘persons in household’ was simplified in the multivariable model to express crowding (≥ 5 persons in household) vs no crowding (< 5).

**Table 4:** Associations between severe tuberculosis and origin, socioeconomic status, and comorbidity among women diagnosed with tuberculosis while pregnant or in the post-partum period (cases) and tuberculosis controls, Denmark, 1990–2018 (n = 2,708)

Characteristic	Multivariable logistic regression^a^		
	OR	95% CI	p^b^
**Category^c^**			
Danes	1	Ref	0.9689
Migrants	0.98	0.34–2.42	
**Charlson comorbidity index**			
0	1	Ref	0.2688
≥ 1	0.68	0.34–1.34	
**Employment status**			
Employed	1	Ref	0.8183
Not-employed	0.87	0.28–2.75	
**Yearly income (EUR) **			
< 15,000	1	Ref	0.1792
≥ 15,000	0.34	0.07–1.63	

^a^ In the final multivariable model there has been adjusted for all the listed variables.

^b^ Wald test.

^c^ The category is based on the geographical area of origin.

## Discussion

In this nationwide retrospective case–control study of women diagnosed with TB during pregnancy or in the post-partum period, the vast majority of such women were migrants recently arriving from TB high-burden countries without significant comorbidities and a TB disease severity similar to non-pregnant women with TB. The fact that cases had shorter median time in Denmark since immigration until TB diagnosis compared with TB controls (2.74 vs 3.98 years), may reflect how pregnancy accelerates progression from TBI to active TB.

Since the 1980s, the TB incidence has declined among native Danes whereas the proportion of migrants among all TB cases has steadily increased now constituting approximately two-thirds of cases [20]. In our study, we found an even higher proportion of migrants, accounting for 92% of cases. The majority were migrant women from Africa among whom 87% (199/228) were Somalis. In line with our findings, a study from the UK found TB during pregnancy limited to ethnic minority women, most commonly recent migrants [22]. Likewise, a study from the United States (US) found a higher proportion of ethnic minorities, such as Hispanic women, with TB during pregnancy [2]. In the 1990s, Denmark experienced a large arrival of migrants from Somalia due to civil war. This arrival is evident in the TB statistics (Figure 2). Therefore, variations in TB cases’ origin will also reflect the TB risk in pregnancy. The composition of migrants will change over time and be affected by wars, political conflicts and natural disasters that may force people to flee from their homelands. This diversity of migrants should be considered when planning control interventions addressing pregnant migrants.

In this study, few cases were employed, and a high proportion received cash benefits (59%). However, in line with publicly available data, female migrants from non-European countries are more frequently recipients of social welfare benefits than Danish women [23]. Though our case population was quite young (one-third below 25 years of age), the educational level was still remarkably low as was the income compared with the pregnant/post-partum controls, reflecting a vulnerable group in the society.

In this study, most TB cases were diagnosed during pregnancy, in contrast to findings from the UK [12] and Sweden [11] who observed the highest incidences in the post-partum period. The fact that many pregnancy-associated TB cases are diagnosed after they give birth rather than during pregnancy could be due to a more conservative approach to investigations during pregnancy [12,24,25], but also because TB symptoms can be misinterpreted as pregnancy-related [2,23,25]. Diagnostic delay can potentially cause more severe disease manifestations. A study from the US found significantly higher maternal mortality among all women with TB during pregnancy relative to controls, attributed to delayed diagnosis and treatment [2]. In the current study however, we found no signs of more severe TB manifestations among cases compared with TB controls and, in addition, there was no increased maternal mortality among cases compared with controls.

Previous studies have demonstrated that in Europe, migrants from Africa and Asia have a higher risk of adverse pregnancy outcomes, though conflicting data exist [26-30]. In Denmark, the antenatal care programme is free to all women with legal residency [31] and it consists of consultations with a doctor and a midwife, antenatal and parent preparation classes, invitations to blood tests, and two ultrasound scans performed to provide early risk assessment and to test for congenital malformations [32]. This comprehensive programme could be an ideal opportunity to perform systematic TB screening in selected women at risk of TB. Testing for HIV and hepatitis is already part of the Danish standard antenatal programme [32]. In Sweden, the Public Health Agency recommends TB screening among pregnant women from high-incidence countries or with known exposure [33]. A recent study evaluated the yield of the new screening programme in Sweden [13], and interestingly, 22% of screened pregnant women from TB high-incidence countries were Interferon Gamma Release Assays (IGRA) positive and a high proportion completed preventive treatment [13], thereby substantially lowering the risk of later TB due to reactivation. Like Sweden, the Centers for Disease Prevention and Control (CDC) in the US recommends screening of all pregnant women with increased risk of TB at the beginning of antenatal care [34]. As the majority of the migrant mothers in our study were recent migrants, several cases could possibly have been prevented through a ‘TB pre-entry screening’ on arrival to Denmark. However, screening for TBI and TB are not systematically offered to all migrant groups [35]. Therefore antenatal care appointments constitute an alternative and promising opportunity to provide TB screening to women from TB high-burden countries. This may also apply to other high-income TB low-incidence countries.

Our study has three major strengths: the nationwide design, long study period, and comprehensive nationwide registers with individually-linked data through the unique CPR number. We have minimised the risk of selection bias by including all cases of TB diagnosed when pregnant and in the post-partum period during the study. However, there are some limitations. The pregnant/post-partum controls were systematically included from a prior-selected control group containing all controls matched individually on sex and age to TB cases, therefore the controls were not entirely uncorrelated with the pregnant/post-partum TB cases (Figure 1). The risk of misclassified cases appearing among controls is very small in this study, because TB in Denmark is rare and TB during pregnancy even more rare. The vast majority of our cases in this study were migrants from TB high-incidence countries. There may be undiagnosed/never reported cases of TB among pregnant migrants e.g. undocumented migrants not having a CPR number, which would result in underestimating the TB risk among pregnant migrants, however we consider this to not affect the final risk estimates and therefore to not change our main findings and conclusion. By including all possible TB cases and all possible pregnancies in the unique Danish register-data-system and by having population controls reflecting the origin and SES distribution in Denmark, we believe that our data are generalisable. Although we adjusted for potential confounders in the regression analyses, there remains a risk of residual confounding from unmeasured or not included factors such as increased risk of exposure in certain environments or increased risk of reactivation of TBI due to risky behaviour e.g. homelessness and drug misuse, or other conditions not associated with pregnancy. Such residual confounding could lead to an overestimation of the pregnancy-related TB risk. The retrospective observational design relies on existing register information leaving the possibility of information bias. The data on education and employment are not complete as many migrants arrived after finishing school in their home countries or arrived recently and were not yet employed. Furthermore, cases were relatively young and could have achieved higher education, employment and income later in life. Nonetheless, this would apply to both cases and controls and should not affect the final estimates. We did not review medical files which might have provided additional information e.g. on possible diagnostic delays and/or clinical symptoms. Thus, the severity of TB discussed is this study is based on proxies for severe disease manifestations.

## Conclusions

Tuberculosis among pregnant and post-partum women primarily occurs among migrants during the first 3 years after arrival to Denmark. Pregnant women with TB constitute a vulnerable group as TB may cause adverse events among mothers and their newborn children. Therefore, we recommend an increased focus on this population and suggest to evaluate targeted TBI and active TB screening of selected at-risk pregnant women, which may prevent future cases.
